# IL-27 increases energy storage in white adipocytes by enhancing glucose uptake and fatty acid esterification

**DOI:** 10.1080/21623945.2023.2276346

**Published:** 2023-11-10

**Authors:** Chiara Scaffidi, Annie Srdic, Daniel Konrad, Stephan Wueest

**Affiliations:** a Division of Pediatric Endocrinology and Diabetology, University Children’s Hospital, University of Zurich, Zurich, Switzerland; b Children’s Research Center, University Children’s Hospital, University of Zurich, Zurich, Switzerland; c Zurich Center for Integrative Human Physiology, University of Zurich, Zurich, Switzerland

**Keywords:** Free fatty acid, triglyceride, lipolysis, obesity, white adipose tissue

## Abstract

The cytokine interleukin (IL)-27 has been reported to induce thermogenesis in white adipocytes. However, it remains unknown whether IL-27-mediated adipocyte energy dissipation is paralleled by an elevated energy supply from lipids and/or carbohydrates. We hypothesized that IL-27 increases lipolysis and glucose uptake in white adipocytes, thereby providing substrates for thermogenesis. Unexpectedly, we found that treatment of 3T3-L1 adipocytes with IL-27 reduced intra- and extracellular free fatty acid (FFA) concentrations and that phosphorylation of hormone-sensitive lipase (HSL) was not affected by IL-27. These results were confirmed in subcutaneous white adipocytes. Further, application of IL-27 to 3T3-L1 adipocytes increased intracellular triglyceride (TG) content but not mitochondrial ATP production nor expression of enzymes involved in beta-oxidation indicating that elevated esterification rather than oxidation causes FFA disappearance. In addition, IL-27 significantly increased GLUT1 protein levels, basal glucose uptake as well as glycolytic ATP production, suggesting that increased glycolytic flux due to IL-27 provides the glycerol backbone for TG synthesis. In conclusion, our findings suggest IL-27 increases glucose uptake and TG deposition in white adipocytes.

## Introduction

Interleukin (IL)-27 is a cytokine with cell and infectious context-dependent pro and anti-inflammatory effects [1,2]. It is a member of the IL-12 family and is comprised of two subunits, Epstein-Barr virus-induced gene 3 (EBI-3) and p28. While immune cells are the primary producers of IL-27, it can also be synthesized by non-immune cells such as adipocytes [2,3]. In the latter, pro-inflammatory factors known to be elevated in obesity, such as tumour necrosis factor alpha (TNFα) and lipopolysaccharides (LPS) increase its release [3–5]. Accordingly, expression of EBI-3 and p28 was found to be increased in white adipose tissue (WAT) of obese mice [6]. In turn, IL-27 may activate stress pathways like extracellular signal-regulated kinase (ERK), signal transducer and activator of transcription (STAT)-3 and p38 mitogen-activated protein kinase (MAPK) in pre-adipocytes, adipocytes or immune cells residing in WAT [2,3]. Since activation of these pathways impacts inflammatory processes and/or lipolysis in WAT [7–11], obesity-induced IL-27 production in white adipocytes may contribute to metabolic dysregulation therein.

However, concentrations of circulating IL-27 are reduced in human subjects with obesity (~700 pg/ml in lean *vs*. ~200 pg/ml in obese). Moreover, IL-27 was identified as a promising target for anti-obesity therapy, since it increases thermogenesis in white adipocytes thereby reducing body weight gain [12]. In particular, IL-27 administration has been shown to increase uncoupling protein 1 (UCP1) levels in a p38-dependent manner, thereby increasing energy dissipation. Of note, UCP1-dependent uncoupling is activated by free fatty acids (FFA) emerging from elevated lipolysis [13,14]. Adipose triglyceride lipase (ATGL) and hormone sensitive lipase (HSL) are the major enzymes contributing to hydrolytic cleavage of FFA from triacylglycerol (TG) [8,15]. Once activated, UCP1 increases glucose and FFA utilization uncoupled from ATP synthesis resulting in energy dissipation [16]. Accordingly, elevated UCP1-dependent uncoupling may be fuelled by energy obtained from lipids and/or carbohydrates. In support of such a notion, UCP1-overexpression in white adipocytes increases glycolytic flux, as indicated by elevated glucose uptake and glycolysis [17]. However, it remains unknown whether IL-27-mediated energy dissipation [12] is paralleled by elevated energy supply from lipids and/or carbohydrates. Herein, we examined whether IL-27 increases lipolysis as well as glucose uptake in white adipocytes, thereby providing substrates for thermogenesis.

## Results

### IL-27 activates stress signalling pathways in white adipocytes

**Figure 1.**
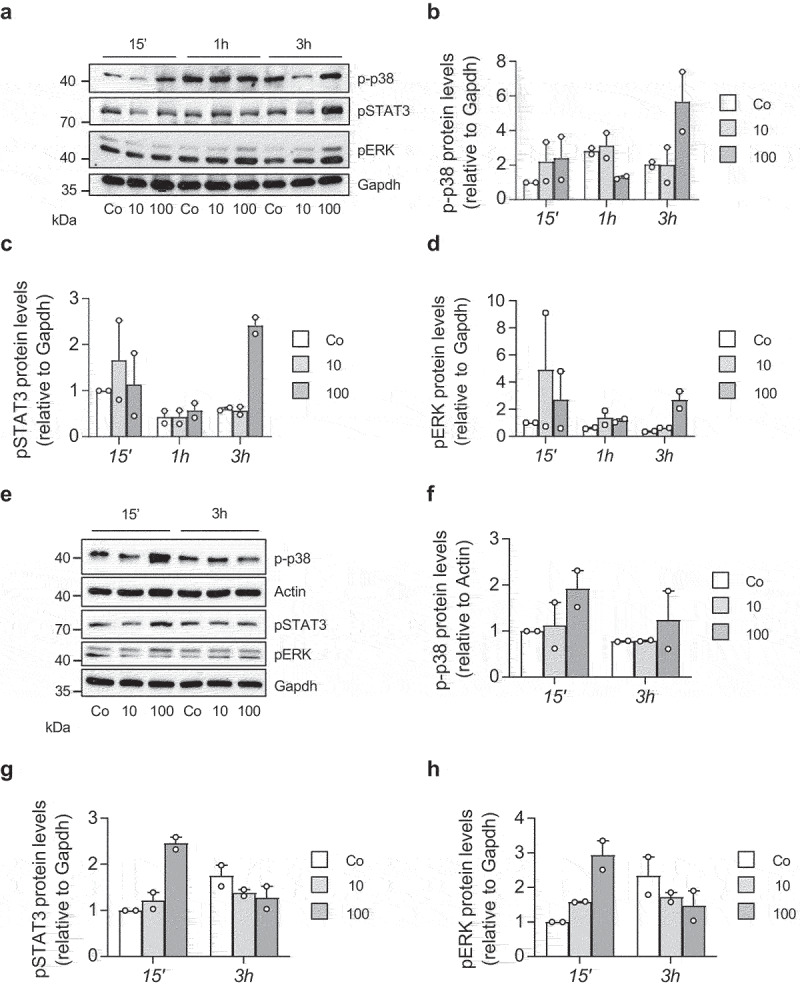
IL-27 activates stress signalling pathways in white adipocytes.

To address whether IL-27 activates stress signalling pathways, mature 3T3-L1 adipocytes were exposed to 10 or 100 ng/ml recombinant IL-27, reflecting a concentration range that was previously used *in vitro* [12,18,19]. As depicted in Figure 1a–d, 100 ng/ml of recombinant IL-27 activated p38 after 15 minutes and 3 hours, whereas ERK and STAT3 were activated after 3 hours, as indicated by increased phosphorylated protein levels. Moreover, application of 100 ng/ml IL-27 resulted in increased phosphorylated p38, pERK and pSTAT3 after 15 minutes in a mouse-derived subcutaneous white adipocyte cell line [20] (Figure 1e–h). Hence, IL-27 increases activation of stress signalling pathways in mature 3T3-L1 as well as subcutaneous white adipocytes.

Representative Western blot and quantification of two independent Western blots of phospho(p)-p38, pSTAT3 and pERK protein levels of 3T3-L1 (a-d) and subcutaneous white adipocytes (e-h) treated with vehicle control (Co) or different concentrations (ng/ml) of recombinant IL-27 (10 or 100) for indicated time periods.

### IL-27 decreases FFA concentration in white adipocytes

**Figure 2.**
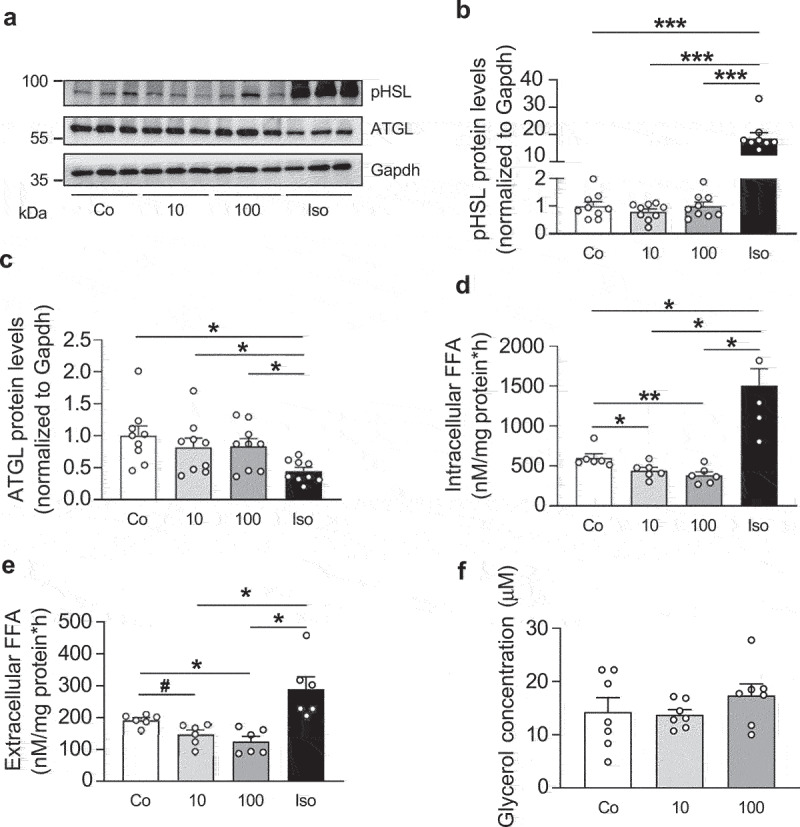
IL-27 decreases FFA concentrations in 3T3-L1 adipocytes.

Since we hypothesized that IL-27 induces lipolysis, its two main regulating enzymes HSL and ATGL were analysed. Phosphorylation of HSL is necessary to induce its translocation to lipid droplets and, hence, its activity; phosphorylation is not needed to activate ATGL [21]. Whereas the positive control isoproterenol increased phosphorylation of HSL as expected, treatment with 10 or 100 ng/ml IL-27 for 3 hours had no effect (Figure 2a,b). In addition, IL-27 had no effect on ATGL protein levels, whereas isoproterenol significantly decreased them (Figure 2a–c), as previously shown [22].

Representative Western blot (a) and quantification (b and c) of ATGL and phospho-HSL protein levels in 3T3-L1 adipocytes treated with vehicle control (Co), different concentrations (ng/ml) of recombinant IL-27 (10 or 100) or 1 µM isoproterenol (Iso) for 4 hours. *n* = 9 biological replicates of three independent experiments. (d and e) Intra and extracellular FFA concentration in mature 3T3-L1 adipocytes treated with vehicle control (Co), different concentrations (ng/ml) of recombinant IL-27 (10 or 100) or 1 µM isoproterenol (Iso) for 4 hours. *n* = 6 biological replicates of two independent experiments. (f) Intracellular glycerol concentration in mature 3T3-L1 adipocytes treated with vehicle control (Co) or different concentrations (ng/ml) of recombinant IL-27 (10 or 100) for 4 hours. *n* = 7 biological replicates of two independent experiments. Data are expressed as mean ± SEM. ^#^p = 0.08, *p < 0.05, **p < 0.01, ***p < 0.001 (ANOVA).

Next, lipolysis was assessed by measuring FFA concentrations in cell lysates (intracellular) as well as in the supernatant (extracellular). Application of both 10 and 100 ng/ml IL-27 significantly decreased concentrations of both intracellular and extracellular FFA (Figure 2d,e). In contrast, IL-27 administration did not affect the intracellular concentration of free glycerol (Figure 2f). As expected, isoproterenol significantly increased FFA concentrations in both cellular compartments (Figure 2d,e).

**Figure 3.**
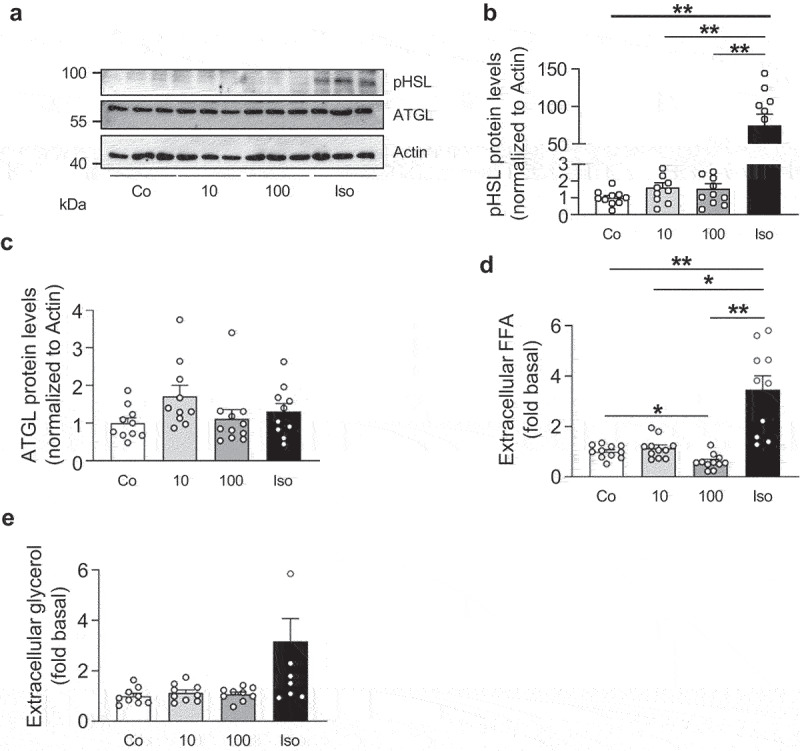
IL-27 decreases FFA concentrations in subcutaneous white adipocytes.

To confirm findings observed in 3T3-L1 adipocytes, we performed experiments in subcutaneous white adipocytes. As reported for 3T3-L1 adipocytes, IL-27 had no effect on HSL phosphorylation and ATGL protein levels (Figure 3a–c). Moreover, 100 ng/ml IL-27 significantly reduced extracellular FFA levels (Figure 3d) without affecting glycerol concentration (Figure 3e). Taken together, IL-27 induces FFA disappearance in white adipocytes without affecting the lipolytic machinery.

Representative Western blot (a) and quantification (b and c) of ATGL and phospho(p)HSL protein levels of subcutaneous adipocytes treated with vehicle control (Co), different concentrations (ng/ml) of recombinant IL-27 (10 or 100) or 1 µM isoproterenol (Iso) for 3 hours. *n* = 10-11 biological replicates of three independent experiments. (d) Extracellular FFA concentration in mature subcutaneous adipocytes treated with vehicle control (Co), different concentrations (ng/ml) of recombinant IL-27 (10 or 100) or 1 µM isoproterenol (Iso) for 4 hours. *n* = 11 biological replicates of four independent experiments. (e) Extracellular glycerol concentration of mature subcutaneous adipocytes treated with vehicle control (Co), different concentrations (ng/ml) of recombinant IL-27 (10 or 100) or 1 µM isoproterenol (Iso) for 4 hours. *n* = 9 biological replicates of three independent experiments. Data are expressed as mean ± SEM. *p < 0.05, **p < 0.01 (ANOVA).

### IL-27 increases triglyceride content in 3T3-L1 adipocytes

**Figure 4.**
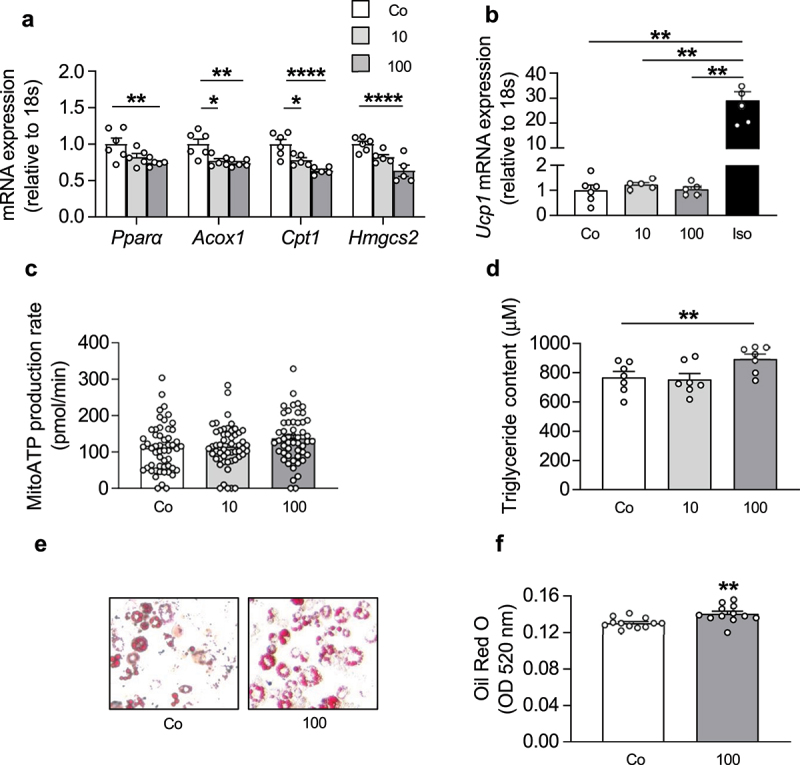
IL-27 increases triglyceride content in 3T3-L1 adipocytes.

In addition to a decrease in lipolysis, reduced FFA concentrations may result from an induction of beta-oxidation or increased re-esterification into TG [13]. As shown in Figure 4a, expression of enzymes involved in beta-oxidation were significantly reduced in 3T3-L1 adipocytes treated with IL-27. Moreover, neither expression of *Ucp1* (Figure 4b) nor mitochondrial ATP production (Figure 4c) was significantly stimulated by IL-27 administration, indicating that neither ATP-uncoupled nor coupled FFA dissipation was elevated. Hence, FFA disappearance is likely caused by increased re-esterification into TG. In line with this, IL-27 treatment significantly increased TG content (Figure 4d) as well as Oil Red O accumulation (Figure 4e,f) in 3T3-L1 adipocytes.

(a) mRNA expression of respective targets in mature 3T3-L1 adipocytes treated with vehicle control (Co) or different concentrations (ng/ml) of recombinant IL-27 (10 or 100) for 4 hours. *n* = 5-6 biological replicates of two independent experiments. (b) *Ucp1* mRNA expression in mature 3T3-L1 adipocytes treated with vehicle control (Co), different concentrations (ng/ml) of recombinant IL-27 (10 or 100) or 1 µM isoproterenol (Iso) for 4 hours. *n* = 5-6 biological replicates of two independent experiments. (c) Mitochondrial (mito)ATP production rate in mature 3T3-L1 adipocytes treated with vehicle control (Co) or different concentrations (ng/ml) of recombinant IL-27 (10 or 100) for 4 hours. *n* = 52-53 biological replicates of two independent experiments. (d) Triglyceride concentration in mature 3T3-L1 adipocytes treated with vehicle control (Co) or different concentrations (ng/ml) of recombinant IL-27 (10 or 100) for 4 hours. *n* = 7 biological replicates of two independent experiments. Oil Red O staining (e) and quantification (f) in mature 3T3-L1 adipocytes treated with vehicle control (Co) or recombinant IL-27 (100 ng/ml) for 4 hours. *n* = 12 biological replicates of two independent experiments. Data are expressed as mean ± SEM. *p < 0.05, **p < 0.01, ***p < 0.001, ****p < 0.0001 (ANOVA for a-d, Student’s t test for f).

### IL-27 increases glucose uptake and glycolysis in 3T3-L1 adipocytes

**Figure 5.**
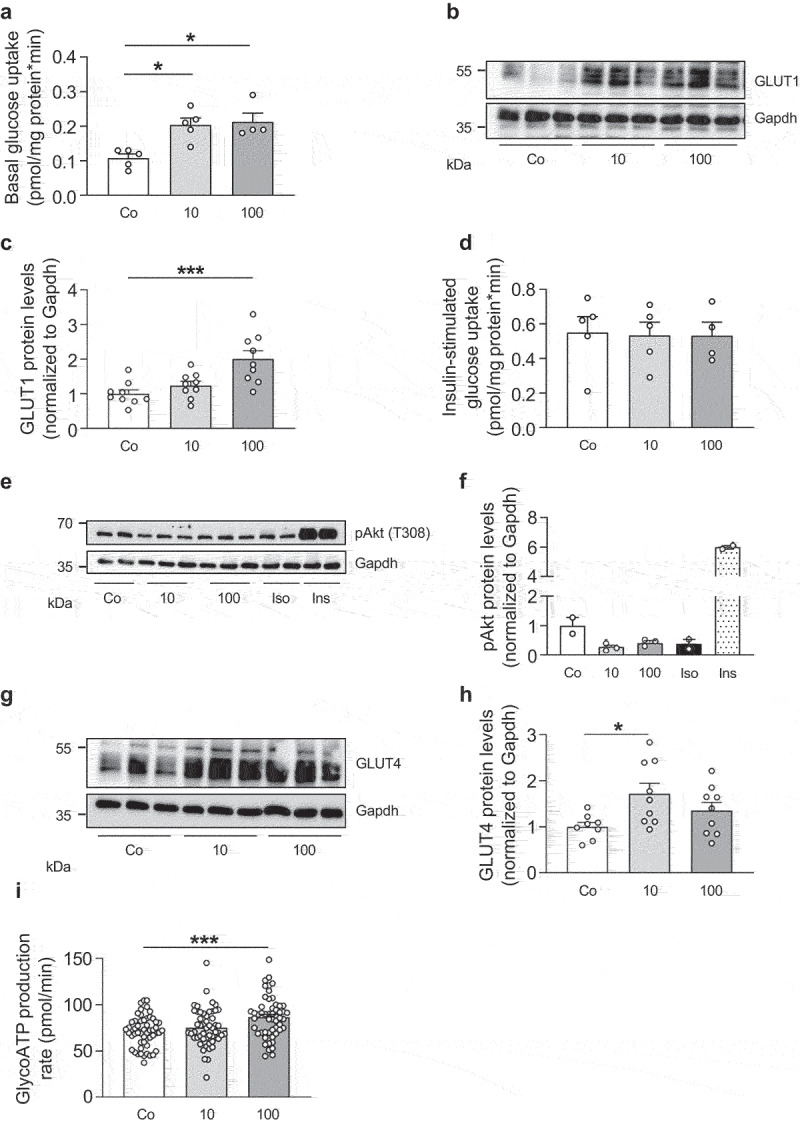
IL-27 increases glucose uptake and glycolysis in 3T3-L1 adipocytes.

Since increased glycolytic flux provides the glycerol backbone of TG, we hypothesized that IL-27 increases glucose uptake thereby increasing glycerol-3-phosphate availability for TG synthesis [23]. As depicted in Figure 5a, IL-27 significantly increased basal glucose uptake into 3T3-L1 adipocytes. This was paralleled by a significant increase in glucose transporter GLUT1 protein levels (Figure 5b,c). In contrast, insulin-stimulated glucose uptake was not altered by IL-27 (Figure 5d). Consistently, IL-27 did not elevate pAkt protein levels in contrast to insulin (Figure 5e,f). Of note, GLUT4 protein levels were significantly higher in adipocytes when treated with 10 ng/ml but not with 100 ng/ml IL-27 (Figure 5g,h). In support for increased glucose flux, IL-27 significantly increased glycolytic ATP production (Figure 5i).

(a) Basal glucose uptake in mature 3T3-L1 adipocytes treated with vehicle control (Co) or different concentrations (ng/ml) of recombinant IL-27 (10 or 100) for 4 hours. *n* = 4-5 biological replicates of five independent experiments. Representative Western blot (b) and quantification (c) of GLUT1 protein levels of 3T3-L1 adipocytes treated with vehicle control (Co) or different concentrations (ng/ml) of recombinant IL-27 (10 or 100) for 4 hours. *n* = 9 biological replicates of three independent experiments. (d) Insulin-stimulated glucose uptake in mature 3T3-L1 adipocytes treated with vehicle control (Co) or different concentrations (ng/ml) of recombinant IL-27 (10 or 100) for 4 hours. *n* = 4-5 biological replicates of five independent experiments. Western blot (e) and quantification (f) of phosphorylated(p) Akt protein levels of 3T3-L1 adipocytes treated with vehicle control (Co), different concentrations (ng/ml) of recombinant IL-27 (10 or 100) for 30 min, 1 µM isoproterenol (Iso) for 30 min or 100 nM insulin (Ins) for 10 min. Representative Western blot (g) and quantification (h) of GLUT4 protein levels of 3T3-L1 adipocytes treated with vehicle control (Co) or different concentrations (ng/ml) of recombinant IL-27 (10 or 100) for 4 hours. *n* = 8-9 biological replicates of three independent experiments. (**i**) Glycolytic (glyco)ATP production rate in mature 3T3-L1 adipocytes treated with vehicle control (Co) or different concentrations (ng/ml) of recombinant IL-27 (10 or 100) for 4 hours. *n* = 53 biological replicates of two independent experiments. Data are expressed as mean ± SEM. *p < 0.05, ***p < 0.001 (ANOVA).

## Discussion

IL-27 is a pleiotropic cytokine with well-known pro- and anti-inflammatory effects in different immune cells [1,2]. However, while it has been known for a decade that IL-27 and its receptor are expressed in WAT and, particularly, in white adipocytes [3,6], its (patho)physiological role has only recently been addressed. Importantly, IL-27 was found to increase thermogenesis in white adipocytes [12]. In the current study, we hypothesized that increased thermogenesis is fuelled by elevated lipolysis and glucose uptake. Unexpectedly, we found that IL-27 decreased FFA concentrations in white adipocytes without affecting the two main lipolytic enzymes HSL and ATGL. Our observation that free glycerol concentration was not changed further suggests IL-27 does not regulate lipolysis. Such notion is based on the fact that glycerol arising from lipolysis is not re-used for TG synthesis, since glycerol kinase is poorly expressed in white adipocytes unless activated by thiazolidinediones [24,25]. Hence, while the concentration of glycerol may be indicative of the rate of lipolysis, changes in FFA concentrations are not only modulated by lipolysis but also by beta-oxidation and re-esterification [13]. In the latter, FFA are esterified to glycerol-3-phosphate emerging from increased glycolysis [23]. Strikingly, IL-27-mediated decreases in FFA concentrations were paralleled by increased glycolysis and TG content, indicating that FFA disappearance resulted from increased re-esterification.

An IL-27-mediated increase in adipocyte lipid storage may prevent ectopic fat accumulation. Importantly, a better understanding of pathways stimulating energy storage in WAT is of interest, since the development of non-alcoholic fatty liver disease (NAFLD) may be caused by constrained lipid storage in WAT leading to systemic lipid overflow and, consecutively, fatty liver disease [26,27]. NAFLD is the most common cause of liver dysfunction and signifies, together with associated obesity and type 2 diabetes, a severe economic and social burden on today’s society [28,29]. In line with a role of IL-27 in preventing the development of NAFLD, administration of IL-27 reduced hepatic lipid accumulation in high fat diet-fed mice [30].

Previously, IL-27 administration increased basal pAkt protein levels in WAT [12], suggesting that IL-27 induces insulin signalling and/or increases insulin sensitivity. Since IL-27 and its receptor are not only expressed in adipocytes but also in other cells residing in WAT, paracrine interactions between immune cells and adipocytes may have contributed to this effect. To avoid paracrine interactions as well as the use of animals to meet the principles of the 3Rs, experiments here were performed in cultured white adipocytes. We found that IL-27 did not increase pAkt protein levels in adipocytes suggesting that IL-27 does not directly activate insulin signalling. Moreover, insulin-stimulated glucose uptake into 3T3-L1 adipocytes was not affected by IL-27. These data suggest that (acute) IL-27 treatment does not affect adipocyte insulin sensitivity. In contrast, IL-27 significantly increased basal glucose uptake as well as protein levels of the glucose transporters GLUT1 and GLUT4. IL-27-induced increase in basal glucose uptake is likely mediated by the elevated GLUT1 since the majority of GLUT1 is distributed to the plasma membrane, whereas GLUT4 under basal conditions is predominantly located within the cytoplasm and only after insulin stimulation translocates to the plasma membrane [31]. The lacking difference in insulin-stimulated glucose uptake indicates that IL-27 does not affect GLUT4 abundance in the plasma membrane despite higher total GLUT4 protein levels.

Activation of the highly thermogenic brown adipose tissue (BAT) is characterized by increased glucose uptake. After entering brown adipocytes via GLUT1, glucose is mainly incorporated into TG, which subsequently serve as an energy substrate for UCP1-mediated thermogenesis [32,33]. Possibly, IL-27 ‘activates’ white adipocytes in a similar way, leading to elevated GLUT1-dependent glucose uptake thereby increasing glycolysis and TG deposition. In turn, TG may be dissipated by IL-27-induced upregulation of UCP1 [12]. The fact that our treatment of 3T3-L1 adipocytes with IL-27 for 4 hours did not increase *Ucp1* expression is in contrast to the observation in primary adipocytes [12]. Similarly, extension of the incubation period to the one used in the latter study (24 hours) did not increase *Ucp1* expression in 3T3-L1 adipocytes (data not shown). We currently have no explanation for these contradicting findings.

In conclusion, IL-27 increases glucose uptake and TG deposition in white adipocytes.

## Methods

### Cell culture

Pre-adipocytes were seeded in tissue culture plates coated with 0.1% gelatin (Millipore, Burlington, MA, USA) and grown until confluency at 37°C in a humidified atmosphere with 5% CO2 in complete medium (CM) containing 4.5 g/L glucose DMEM (ThermoFisher Scientific, Waltham, MA, USA), 10% foetal bovine serum, 100 IU/ml penicillin and 100 μg/ml streptomycin. Differentiation of 3T3-L1 cells was initiated two days after reaching confluency (day 0) using CM supplemented with 500 μM 3-isobutyl-1-methylxanthine (IBMX, ThermoFisher Scientific), 1 μM dexamethasone, 1.7 μM human insulin (Actrapid, Novo Nordisk, Bagsværd, Denmark) and 1 μM rosiglitazone (ThermoFisher Scientific). On day 3, cells were switched to CM containing 0.5 μM insulin for 48 hours (day 5). To complete differentiation, cells were cultured in CM with 2% FBS until day 7 and from here onwards maintained on CM containing 1 g/L glucose DMEM (ThermoFisher Scientific) and 2% FBS for two days. Subcutaneous pre-adipocytes were differentiated using a similar protocol until day 5 and from then onwards maintained on 10% FBS medium as previously described [34]. Mature adipocytes were treated with recombinant murine IL-27 (Biolegend, San Diego, CA, USA) or vehicle control (PBS supplemented with 1% BSA) as indicated.

### Lipolysis

Mature 3T3-L1 or subcutaneous adipocytes were cultured for a total of 4 hours in the presence or absence of 10 or 100 ng/ml recombinant IL-27 or 1 µM isoproterenol Sigma-Aldrich (Merck Group, St. Louis, MO, USA). During the last 3 hours, CM was replaced by IL-27 or isoproterenol containing Krebs Ringer phosphate-HEPES buffer (KRB) supplemented with 0.1% fatty acid-free BSA after a brief rinse in warm PBS. FFA concentration in KRB was determined using a WAKO kit (Fujifilm, Tokyo, Japan) and normalized to total protein content. Glycerol was determined using a kit (F6428) from Sigma-Aldrich.

### Glucose uptake

3T3-L1 adipocytes were seeded (25’000/well) onto gelatin-coated 24-well plates and differentiated as described above. Subsequently, mature adipocytes were treated with or without 10 ng/ml or 100 ng/ml IL-27 for 4 hours. After washing twice with HEPES-buffered saline (140 mM NaCl, 20 mM Na-HEPES, 2.5 mM MgSO4, 1 mM CaCl2, 5 mM KCl, pH 7.4), cells were incubated for 5 min in HEPES-buffered saline containing 10 μM unlabelled 2-deoxyglucose and 0.2 μCi/ml 2-deoxy-[14C]glucose (PerkinElmer, Waltham, MA, USA). The reaction was terminated by washing three times with ice-cold 0.9% NaCl. Non-specific uptake was determined in the presence of 25 μm cytochalasin B. Radioactivity was determined by liquid scintillation counting after lysing cells in 0.05 N NaOH. Counts were normalized to protein content determined using the Pierce BCA Protein Assay Kit (ThermoFisher Scientific).

### Intracellular glycerol and triglyceride determination

Intracellular free glycerol concentration and triglyceride content was determined using the Triglyceride-Glo™ Assay (Promega, Madison, WI, USA) according to manufacturer’s instruction. In brief, cell lysates were treated with or without lipase to determine triglyceride or free glycerol concentration, respectively. Glycerol was measured in a coupled reaction linking glycerol concentration to luciferin, which is detected in a luciferase reaction using Ultra-Glo™.

### Oil red O staining

After rinsing with PBS, cells were fixed in 10% wt/vol. formaldehyde solution (AppliChem, Darmstadt, Germany) for 30 minutes, rinsed in dH_2_O and dehydrated in 60% vol./vol. isopropanol for 4 min. Subsequently, cells were stained with 0.3% wt/vol. Oil-Red-O (O0625; Sigma-Aldrich, Buchs, Switzerland) dissolved in 100% isopropanol that was freshly diluted in dH_2_O to 60% vol./vol. After 5 minutes, cells were rinsed three times with tap water and imaged using an Axio Observer microscope platform (Zeiss, Jena, Germany). Oil Red O was extracted by adding 100% isopropanol for 30 minutes while shaking the plate, and optical density (OD) was measured at 520 nm.

### Mitochondrial respiration and glycolysis

Cells were seeded (7’000/well) onto gelatin-coated 96-well Seahorse cell culture microplates and differentiated as described above. Mature adipocytes were treated with or without IL-27 for 3 hours. Subsequently, medium was replaced by Seahorse XF DMEM Medium pH 7.4 supplemented with 5.5 mM IL-27, 4 mM glutamine, 1 mM pyruvate and 2% FFA-fed BSA. The plate was degassed in a non-CO_2_ incubator at 37°C for 1 hour. After measuring basal oxygen consumption rate (OCR) and extracellular acidification rate (ECAR) in a Seahorse XF Pro Extracellular Flux Analyser (Agilent Technologies, Santa Clara, CA, USA), cells were sequentially treated with Oligomycin (5 µM) and Antimycin A (5 µM). ATP production from glycolysis (glycoATP) and mitochondria (mitoATP) was calculated using Seahorse Analytics software (Agilent).

### Protein isolation and Western blotting

CProteins were extracted in RIPA buffer supplemented with phosphatase and proteinase inhibitors. Protein concentration was determined with the Pierce BCA Protein Assay Kit (ThermoFisher Scientific). Details on Western blotting procedures were previously described [34]. In brief, equal quantities of protein were resolved using SDS-PAGE and transferred onto nitrocellulose membranes, blocked in 5% dry fat milk, incubated with primary antibodies at 4°C and subsequently with corresponding secondary antibodies at room temperature. Developed membranes were imaged with a ChemiDoc MP Imaging System (BioRad, Hercules, CA, USA) and quantified with ImageLab software (BioRad, version 5.2.1). The following primary antibodies were used (diluted 1:1’000 except Actin, which was diluted 1:5’000): pERK1/2, #9101; p-p38, #9212, pSTAT3, #9145; ATGL, #2138; pHSL #4139 (all from Cell Signaling); Gapdh, G9545 (Sigma-Aldrich), Actin, MAB1501 (Millipore), GLUT1 and GLUT4 (gift from Dr. A. Klip, The Hospital for Sick Children, Toronto, ON, Canada; samples were not boiled for Western blots assessing GLUT1 and GLUT4 protein levels).

### RNA extraction and quantitative real-time PCR

Total RNA was extracted with the NucleoSpin® RNA isolation kit (Macherey-Nagel, Düren, Germany). RNA concentration was determined using NanoDrop® spectrophotometer (ThermoFisher Scientific). Equal amounts of RNA were reverse-transcribed with the GoScript™ Reverse Transcription System (Promega) and cDNA amplified by TaqMan real-time PCR using the following probes/primers: Pparα, Mm00627559_m1; Acox1, Mm00443579_m1; Cpt1, Mm00550438_m1; Hmgcs2, Mm00550050_m1; Ucp1 Mm01244861_m1; 18s 4,352,930 (Applied Biosystems, Rotkreuz, Switzerland). Relative gene expression values were obtained after normalization to 18s using the 2^−ΔΔCt^ method.

### Data analysis

Data are presented as means ± SEM and were analysed by two-tailed Student’s *t* test, one-way or two-way ANOVA with Tukey multiple comparisons. Statistical tests were calculated using GraphPad Prism 8.00 (GraphPad Software, San Diego, CA, USA). *p* values < 0.05 were considered to be statistically significant.

### Data availability

All data supporting the findings of this study are available within the article, its Supplementary Figures or under https://zenodo.org/record/8362627.

## Supplementary Material

Supplemental Material
