# Supplementary material for: IL-27 increases energy storage in white adipocytes by enhancing glucose uptake and fatty acid esterification
Source: Adipocyte. 2023 Nov 10;12(1):2276346. doi: 10.1080/21623945.2023.2276346 (PMC10773535; doi:10.1080/21623945.2023.2276346)
Supplement: Supplemental Material [file KADI_A_2276346_SM5476.docx]

# IL-27 increases energy storage in white adipocytes by enhancing glucose uptake and fatty acid esterification

Chiara Scaffidi, Annie Srdic, Daniel Konrad, Stephan Wueest

# Appendix

**Supplementary Figures 1-4**

1

15’ 3h

40

40

70

40

35

kDa

Co 10

100 Co

10 100

p-p38 Actin

pSTAT3 pERK GAPDH


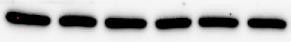

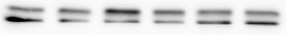

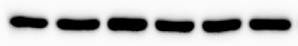

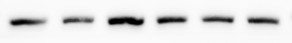

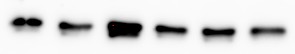


**Supplementary Fig. 1 IL-27 activates stress signaling pathways in subcutaneous white adipocytes** Western blot of phospho(p)-p38, pSTAT3 and pERK protein levels of subcutaneous adipocytes treated with vehicle control (Co) or different concentrations (ng/ml) of recombinant IL-27 (10 or 100) for indicated time periods.

3

Glycerol concentration (μM)

2

1

0

Co 10 100

## Supplementary Fig. 2 IL-27 does not affect intracellular free glycerol concentration

Intracellular glycerol concentration in mature 3T3-L1 adipocytes treated with vehicle control (Co) or different concentrations (ng/ml) of recombinant IL-27 (10 or 100) for 4 hours. n=7 biological replicates of 2 independent experiments. Data are expressed as mean ± SEM.

# a

100

| 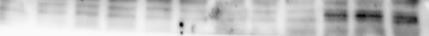 |
| --- |
| 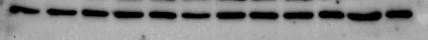 |
|  |


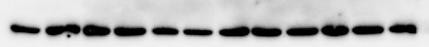
55

40

Co

kDa

10 100 Iso

pHSL ATGL

Actin

**b**

150

pHSL protein levels (normalized to Actin)

100

50

3

2

1

0

**~~**~~**

**** ****

Co 10 100 Iso

# c

**d**


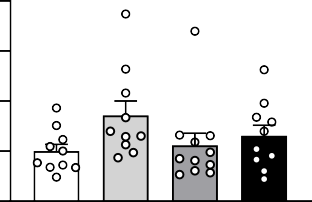
4

ATGL protein levels (normalized to Actin)

3

2

1

0

Co 10 100 Iso

6


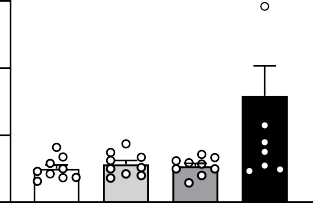
4

Extracellular glycerol (fold basal)

2

0

Co 10 100 Iso

**Supplementary Fig. 3 IL-27 does not affect lipolytic enzymes in subcutaneous white adipocytes** Representative Western blot (**a**) and quantification (**b** and **c**) of ATGL and phospho(p)HSL protein levels of subcutaneous adipocytes treated with vehicle control (Co), different concentrations (ng/ml) of recombinant IL-27 (10 or 100) or 1 µM isoproterenol (Iso) for 3 hours. n=10-11 biological replicates of 3 independent experiments. (**d**) Extracellular glycerol concentration of mature subcutaneous adipocytes treated with vehicle control (Co), different concentrations (ng/ml) of recombinant IL-27 (10 or 100) or 1 µM isoproterenol (Iso) for 4 hours. n=9 biological replicates of 3 independent experiments. Data are expressed as mean ± SEM. **p<0.01 (ANOVA).

# a

Insulin-stimulated glucose uptake (pmol/mg protein*min)

0.8

0.6

0.4

0.2

0.0

Co 10 100

**b**

70


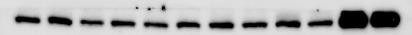

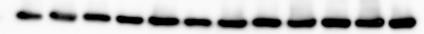


35

kDa Co 10

100 Iso Ins

pAkt (T308) Gapdh

# c

55


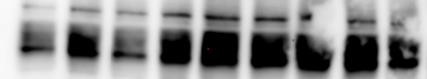

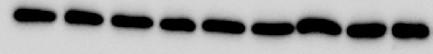


35

Co

kDa

10 100

GLUT4

Gapdh

3

2


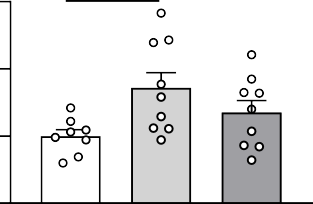


*****

GLUT4 protein levels (normalized to Gapdh)

1

0

Co 10 100

## Supplementary Fig. 4 IL-27 does not affect insulin-stimulated glucose uptake in 3T3-L1 adipocytes

(**a**) Insulin-stimulated glucose uptake in mature 3T3-L1 adipocytes treated with vehicle control (Co) or different concentrations (ng/ml) of recombinant IL-27 (10 or 100) for 4 hours. n=4-5 biological replicates of 5 independent experiments. (**b**) Western blot of phosphorylated(p) Akt protein levels of 3T3-L1 adipocytes treated with vehicle control (Co), different concentrations (ng/ml) of recombinant IL-27 (10 or 100) for 30 min, 1µM isoproterenol (Iso) for 30 min or 100 nM insulin (Ins) for 10 min. Representative Western blot (**c**) and quantification (**d**) of GLUT4 protein levels of 3T3-L1 adipocytes treated with vehicle control (Co) or different concentrations (ng/ml) of recombinant IL-27 (10 or 100) for 4 hours. n=8-9 biological replicates of 3 independent experiments. Data are expressed as mean ± SEM. *p<0.05 (ANOVA).
